# Ten-year follow-up of degenerative spinal lesions on radiographs and MRI in axial spondyloarthritis: results of the DESIR (DEvenir des spondylarthropathies indifférenciées récentes) cohort

**DOI:** 10.1007/s00330-025-11432-4

**Published:** 2025-03-06

**Authors:** Laura Pina Vegas, Miranda van Lunteren, Damien Loeuille, Caroline Morizot, Esther Newsum, Sofia Ramiro, Floris van Gaalen, Alain Saraux, Pascal Claudepierre, Antoine Feydy, Désirée van der Heijde, Monique Reijnierse

**Affiliations:** 1 Department of Rheumatology, Leiden University Medical Center, Leiden, The Netherlands; 2 Department of Rheumatology, CHU de Nancy, Nancy, France; 3 Department of Radiology, Nij Smellinghe, Drachten, The Netherlands; 4 Department of Rheumatology, Zuyderland Medical Center, Heerlen, The Netherlands; 5 Université de Bretagne Occidentale (Univ Brest), Department of Rheumatology, CHU Brest, INSERM (U1227), LabEx IGO, Brest, Brest, France; 6 Department of Rheumatology, CHU Henri Mondor, AP-HP, Créteil, France; 7 EpiDermE, Université Paris-Est Créteil Val de Marne, Créteil, France; 8 Department of musculoskeletal Radiology, CHU Cochin, AP-HP, Paris, France; 9 Department of Radiology, Leiden University Medical Center, Leiden, The Netherlands

**Keywords:** Axial spondyloarthritis, Osteoarthritis, Spine, Magnetic resonance imaging, X-rays

## Abstract

**Objectives:**

To investigate the occurrence of spinal degenerative lesions (DL)s in axial spondyloarthritis (axSpA) inception cohort in radiographs and MRI over 10 years (10Y), to assess their changes over time and factors associated with them.

**Methods:**

Whole spine MRI and cervical and lumbar spine radiographs at baseline/5Y/10Y of patients with axSpA from the DESIR cohort were assessed for DLs by three readers. For descriptive analyses, DLs were defined by agreement between ≥ 2/3 readers or using the average of their assessments, at the patient level (≥ 1 lesion/patient). To assess the progression of DLs over time, we used multilevel generalised estimating equation models considering individual reader data.

**Results:**

Imaging was available for 330 patients (mean age 34 [9] years, 47% male). At baseline, 53% of patients had ≥ 1 DL on radiographs and 94% on MRI; 71% and 97% had DL at 10Y, respectively. The most frequent lesion on radiographs was disc height loss (baseline: 45% of patients, 10Y: 65%) and MRI disc degeneration (86%, 95%). Progression over time on radiographs was detected for osteophytes (change/Y: 2.34%, 95% CI: 1.92–2.75), disc height loss (1.37%, 0.95–1.80) and facet joint osteoarthritis (1.30%, 0.90–1.69) and on MRI for disc bulging/herniation (1.19%, 0.74–1.64), Modic type I (1.01%, 0.69–1.33) and II (0.94%, 0.66–1.22) lesions. We also observed a significant increase per year in the total number of DLs on radiographs (β: 1.81, 1.48–2.14) and MRI (β: 4.17, 3.49–4.84). Associated factors in both modalities were increasing BMI and bDMARDs exposure.

**Conclusion:**

In axSpA spinal DLs, though common, progress very slowly over 10Y. Faster progression is observed with increasing BMI and bDMARDs exposure (severe axSpA).

**Key Points:**

***Question*** *The long-term evolution of spinal DLs in axSpA on radiographs and MRI, and the associated factors, is currently poorly understood*.

***Findings*** *Spinal DLs, although common, progress slowly over 10Y, but in patients with a higher BMI or exposed to bDMARDs, the progression is faster*.

***Clinical relevance*** *Understanding the progression of spinal DLs in axSpA helps to refine the interpretation of long-term imaging, limit diagnostic errors and optimise management strategies, particularly in patients with the highest risk of progression of these lesions*.

## Introduction

Axial spondyloarthritis (axSpA) is a chronic inflammatory rheumatic disease mainly affecting the spine, but may also present with peripheral symptoms (joints and entheses), as well as extra-musculoskeletal involvement [1]. Radiographs and MRIs are commonly used to assess both inflammatory and structural lesions. However, they can also provide insight into morphological and even biomechanical changes in the spinal components, and the presence of degenerative lesions (DLs). A high prevalence of spinal DLs has been reported, including in axSpA patients [2, 3]. In a previous study conducted in the DESIR (DEvenir des spondylarthropathies indifférenciées récentes) cohort, almost 30% of patients had spinal DLs on radiographs and over 70% on MRI at baseline [3]. In another inception cohort, SPACE (Spondyloarthritis Caught Early), the prevalence of spinal DLs at baseline was estimated at 44% and 89% respectively [2]. This prevalence was similar in patients without axSpA and in those with possible or definite axSpA, in accordance with the literature [2, 3].

Despite their high initial prevalence, the evolution of DLs in axSpA over the long term remains poorly documented. The general hypothesis is that the number of DLs increases over time, but this has not been established, and it is not known whether some lesions progress faster than others. This question could, however, be of considerable interest to clinical practice. It has been shown that the presence of DLs could delay the diagnosis of axSpA by almost 3 years [4]. Similarly, the change of these lesions over time could lead to a change in the management of axSpA, with a risk of under- or over-treatment in cases of confusion about the origin of imaging lesions in these young patients. The progressive ankylosis that can be observed in axSpA, particularly due to the development of syndesmophytes or facet joint fusion, could contribute to the evolution of DLs. It would lead to a loss of range of motion, a crucial factor in the pathogenesis of degenerative spinal conditions, resulting in alterations to the spinal biomechanics. Furthermore, the factors associated with the progression of DLs in such a population are not known.

Our objectives were (1) to report the occurrence of degenerative spinal lesions over 10 years (10Y) in young patients with axSpA on radiographs and MRI and (2) to assess the change of these lesions as well as the factors associated with them.

## Materials and methods

### Data source and study population

The study was conducted in accordance with Good Clinical Practice guidelines and was approved by the appropriate local medical ethics committees. All patients gave written informed consent on inclusion in the cohort.

For this study, 10Y follow-up data from the DESIR cohort (ClinicalTrials.gov ID:NCT01648907) were used, which has been previously described [5]. Briefly, patients aged 18–50 years from 25 French centres with a clinical diagnosis of axSpA were prospectively included. A detailed description of the study protocol is available at the DESIR website (http://www.lacohortedesir.fr/desir-in-english/) [6, 7]. All patients whose DLs could be assessed at least one-time point during the 10Y were included in these analyses. As DESIR is an inception cohort, some patients were considered not to have axSpA during follow-up and were therefore withdrawn from the study.

### Imaging

Lateral cervical and lumbar radiographs and sagittal T1-weighted turbo spin echo and short-tau inversion recovery (STIR) MRI (1.0–1.5 T, slice thickness of 4 mm) of the whole spine at baseline, 2Y (only for patients who had no data at 5Y)/5Y and 10Y of axSpA patients were used. Three central readers (D.L., E.N., and C.M.) were trained to identify DLs (supervised by M.R., a musculoskeletal radiologist with extensive experience) and assessed each parameter independently, blinded to time, clinical information, and other imaging modalities.

### Radiographic parameters

The following lesions were scored on radiographs [2, 3]. Disc height loss is defined as the narrowing of the disc space relative to two adjacent (healthy) discs. By definition, the lumbar disc spaces should increase in height in the cranial to caudal direction, except disc L5–S1, which normally is smaller than disc L4–L5. Osteophytes are characterised by reactive bone hypertrophy, seen as bony spurs arising from the vertebral body close to the vertebral endplate in a horizontal configuration. Sclerosis is defined as an increased bone density adjacent to the vertebral endplates. Facet joint osteoarthritis (FJOA) is defined as sclerotic joint surfaces and osteophyte formation. Schmorl’s nodes are characterised by a radiolucent contour defect of the vertebral endplate with sclerotic margins [8]. Spondylolisthesis is a condition that occurs when a vertebral body slips relative to the adjacent vertebral body because of spondylolysis (a congenital defect) or FJOA. It is graded according to the percentage of anterior translation with respect to the adjacent level on a three-point scale (grade I: a slip of less than one-third, grade II: a slip of up to two-thirds, grade III: a slip of more than two-thirds) [9].

### MRI parameters

The following parameters are used on MRI [2, 3]. Disc degeneration is scored using the Pfirrmann classification (Pfirrmann class), a five-point scale combining intervertebral disc height loss and signal loss on STIR images [10, 11]. The discs are then categorised as normal (grades ≤ 2) or degenerated (grades > 2). The high-intensity zone (HIZ), indicative of an annular tear or fissure, is defined as an area of high signal intensity located in the posterior annulus fibrosis on STIR images [12, 13]. We considered discs extending beyond the limits of the anatomical disc space as disc bulging or herniation [2, 3]. Canal stenosis (CS) is defined as a reduction of the anterior–posterior diameter of the spinal canal with compression on the spinal cord (cervical/thoracic) or the cauda equina. Neural foraminal stenosis (lateral stenosis) was defined as contact between the nerve root and disc material with obliteration of perineural intraforaminal fat or compression of the nerve root [14–16]. The Modic classification was used to assess DLs of adjacent vertebral endplates. This is a three-point scale, with type I defined as bone marrow oedema (T1: low signal, STIR: high signal), type II defined as fatty changes (T1: high signal, STIR: low signal) and type III defined as sclerotic changes (T1: low signal, STIR: low signal) [17–19]. Schmorl’s nodes are defined as an indentation of the endplate with herniation of intervertebral disc material into the vertebra, with or without oedema [8, 20]. Scheuermann kyphosis/disease, is an excessive spinal curvature, usually in the thoracic spine, involving both vertebral bodies and discs. It is characterised by anterior wedging of ≥ 5 degrees in ≥ 3 adjacent vertebral bodies. We used definitions similar to those of radiographic lesions for FJOA and spondylolisthesis.

### Statistical analysis

For the first part of the study, we aimed to report the spinal DL occurrence over 10Y. Only patients with baseline and 10Y images (radiographs/MRI separately) available, were included in this analysis. Each DL was based on agreement between ≥ 2 out of 3 central readers for binary results and an average of three central readers for continuous results. DLs were reported at both patient (≥ 1 lesion in ≥ 1 vertebral level per patient) and vertebral unit levels using frequency for categorical data and mean and standard deviation (SD) for continuous data. We determined the net percentage change (net progression) between 0Y and 10Y by subtracting the number of patients with a negative change (no specific lesion at 10Y in a patient with ≥ 1 such lesion in ≥ 1 vertebral level at baseline) from those with a positive change (≥ 1 specific lesion in ≥ 1 vertebral level at 10Y in a patient without such lesion at baseline), divided by the total number of patients included in the analysis. We then calculated the number of DLs per patient in the total spine and per segment (cervical, ± thoracic, lumbar spine) at each time point on both imaging modalities. The total DLs number at baseline and 10Y were compared using paired Student’s *t*-test. Finally, we calculated the odds ratio (ORs) and their 95% confidence intervals (95% CI) assessing the relationship between cervical and lumbar spine involvement at baseline and at 10Y on radiographs and MRI.

We assessed the inter-reader reliability among the three readers regarding the total number of DLs at the patient level (patients with baseline and 10Y images) for each imaging modality at baseline and 10Y and regarding 10Y change using the intraclass correlation coefficients (ICC) with a two-way random effects model, using single measures (absolute agreement). ICC values below 0.40 indicate poor reliability, 0.40–0.59 moderate, 0.60-0.74 good and above 0.75 excellent [21].

For the second part of the study, we sought to determine the progression of DLs per imaging modality over time. All patients with DLs assessed at least one-time point were included in these analyses. We used multilevel generalised estimating equation (GEE) models considering individual reader data for each available time point and exchangeable working correlation structure to handle repeated observations over time for each DL and for the total DL number. The main variable of interest was time calculated as the annual progression of each lesion (specifically, the progression in the number of patients with a particular type of DL). Models were adjusted for sex, HLA-B27 status, body mass index (BMI) (kg/m^2^), smoking (ever vs never) and job type (not employed, white or blue collar) at baseline and for biological disease-modifying antirheumatic drugs (bDMARDs) exposure (ever vs never) during the 10Y period. Interactions were tested and if significant (*p* < 0.15), stratified analyses were performed. The percentage of annual change and their 95% CI were reported for dichotomous outcomes and β coefficients and 95% CI were reported for continuous outcomes.

Analyses were performed with R (V4.3.2); a *p* < 0.05 was considered significant.

## Results

**Fig. 1:**
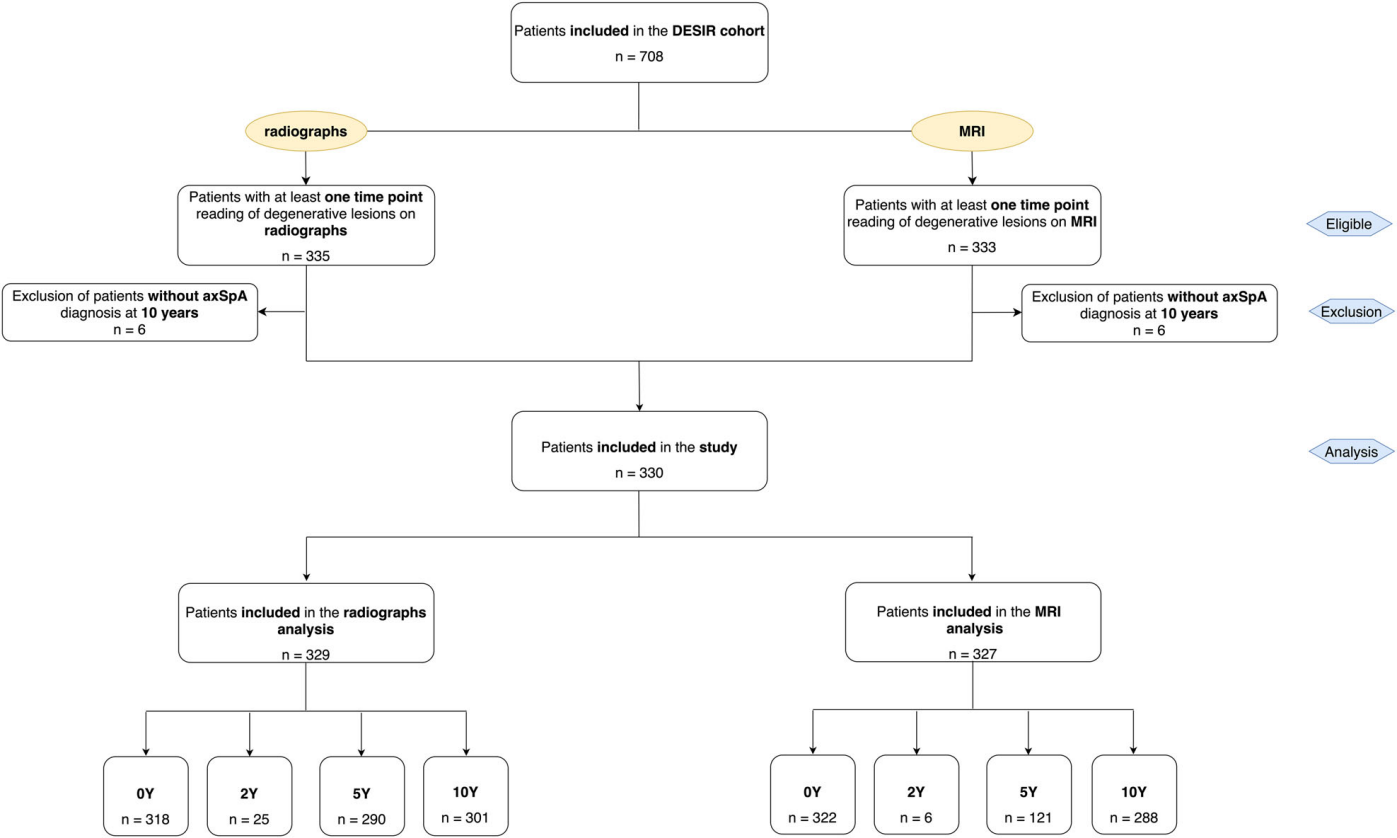
Flow-chart of the patients included in the analysis. Imaging at 2Y was only scored for patients who had no data at 5Y. DESIR, devenir des spondyloarthropathies indifférenciées récentes; MRI, magnetic resonance imaging; axSpA, axial spondyloarthriti; Y, year

After excluding patients without axSpA diagnosis at 10Y (*n* = 6), imaging to assess DLs was available at least one-time point for 330 patients (mean age 34.5 [8.6] years, 47% male): radiographs were available for 329 patients and MRI for 327 patients. Of these, 290 (88%) patients had images at both baseline and 10Y on radiographs; 283 (87%) had an MRI (Fig. 1).

From the 330 patients for whom imaging was available, 66% fulfilled the ASAS (Assessment of SpondyloArthritis International Society) criteria, 64% were HLA-B27-positive, 16% had radiographic sacroiliitis and 30% had MRI active sacroiliitis at baseline (Supplementary Table S1*)*.

### Inter-reader reliability

The ICC associated with the total DLs number, measuring the agreement between the three readers, showed good to excellent reliability for both imaging modalities (0.60–0.76). The ICC associated with the 10Y change in the total number of DLs showed moderate reliability, (0.49–0.51) (Supplementary Table S2).

### Prevalence of degenerative spinal lesions over 10Y

At baseline, 154 (53%) patients had at least one DL on radiographs, increasing to 207 (71%) at 10Y. The most frequent DLs on radiographs were disc height loss (baseline: 45% of patients, 10Y: 65%, net progression: +20%), followed by osteophytes (baseline: 21%, 10Y: 44%, net progression: +22%) and FJOA (baseline: 11%, 10Y: 24%, net progression: +13%) (Table 1). Only 6% (9/158) of patients had osteophytes without disc height loss at baseline; this figure was 8% (8/101) at 10Y. An average total number of 1.6 (2.5) DLs per patient was observed on radiographs at baseline; this number increased to 3.4 (3.9) at 10Y (*p* < 10^−4^). It should be noted that the total number of DLs increased with age.

**Table 1:** DLs observed on radiographs at the patient level (at least one lesion in at least one vertebral level per patient) among patients with images available at baseline and at 10Y

	Baseline	5Y	10Y	Present at least one-time point	10-Year net progression
	*n* = 290	*n* = 258	*n* = 290	*n* = 290	*n* = 290
Loss of disc height	132 (45%)	152 (59%)	189 (65%)	196 (67%)	+20%
Osteophytes	62 (21%)	82 (32%)	127 (44%)	129 (44%)	+22%
FJOA	32 (11%)	45 (17%)	69 (24%)	71 (24%)	+13%
Schmorl’s node	21 (7%)	28 (11%)	29 (10%)	30 (10%)	+3%
Sclerosis	10 (3%)	18 (7%)	28 (10%)	30 (10%)	+6%
Spondylolisthesis	5 (2%)	5 (2%)	10 (3%)	12 (4%)	+2%
Spondylolisthesis grade 1	5 (2%)	5 (2%)	10 (3%)	12 (4%)	+2%
Spondylolisthesis grade 2	0 (0%)	0 (0%)	0 (0%)	0 (0%)	0%
Spondylolisthesis grade 3	0 (0%)	0 (0%)	0 (0%)	0 (0%)	0%
Number of DLs (mean (SD))	1.6 (2.5)	2.5 (3.3)	3.4 (3.9)	–	–
Patients aged < 30 years at baseline (*n* = 100)	0.6 (1.1)	0.8 (1.3)	1.2 (2.2)	–	–
Patients aged 30–40 years at baseline (*n* = 111)	1.2 (2.2)	2.2 (3.1)	3.4 (3.6)	–	–
Patients aged ≥ 40 years at baseline (*n* = 79)	3.3 (3.3)	4.8 (3.8)	6.3 (4.3)	–	–
Number of DLs in the cervical spine (mean (SD))	0.6 (1.3)	1.0 (1.6)	1.5 (2.0)	–	–
Number of DLs in the lumbar spine (mean (SD))	0.9 (1.6)	1.5 (2.2)	1.9 (2.6)	–	–
No DL	136 (47%)	84 (32%)	83 (29%)	–	–

Loss of disc height was defined as the narrowing of the disc space in comparison with two adjacent (healthy) discs. Osteophytes were described by reactive bone hypertrophy, seen as bony spurs arising from the vertebral body close to the vertebral endplate in a horizontal configuration (maximum of 45-degree angle with the endplate). Sclerosis was defined by an increased bone density and calcification adjacent to the vertebral endplates. FJOA was defined as sclerotic joint surfaces and osteophyte formation. Schmorl’s nodes were defined as a radiolucent contour defect of the vertebral endplate with sclerotic margins. Spondylolisthesis was defined as the slippage of one vertebral body with respect to the adjacent vertebral body

*SD* standard deviation, *FJOA* facet joint osteoarthritis, *DL* degenerative lesion

**Fig. 2:**
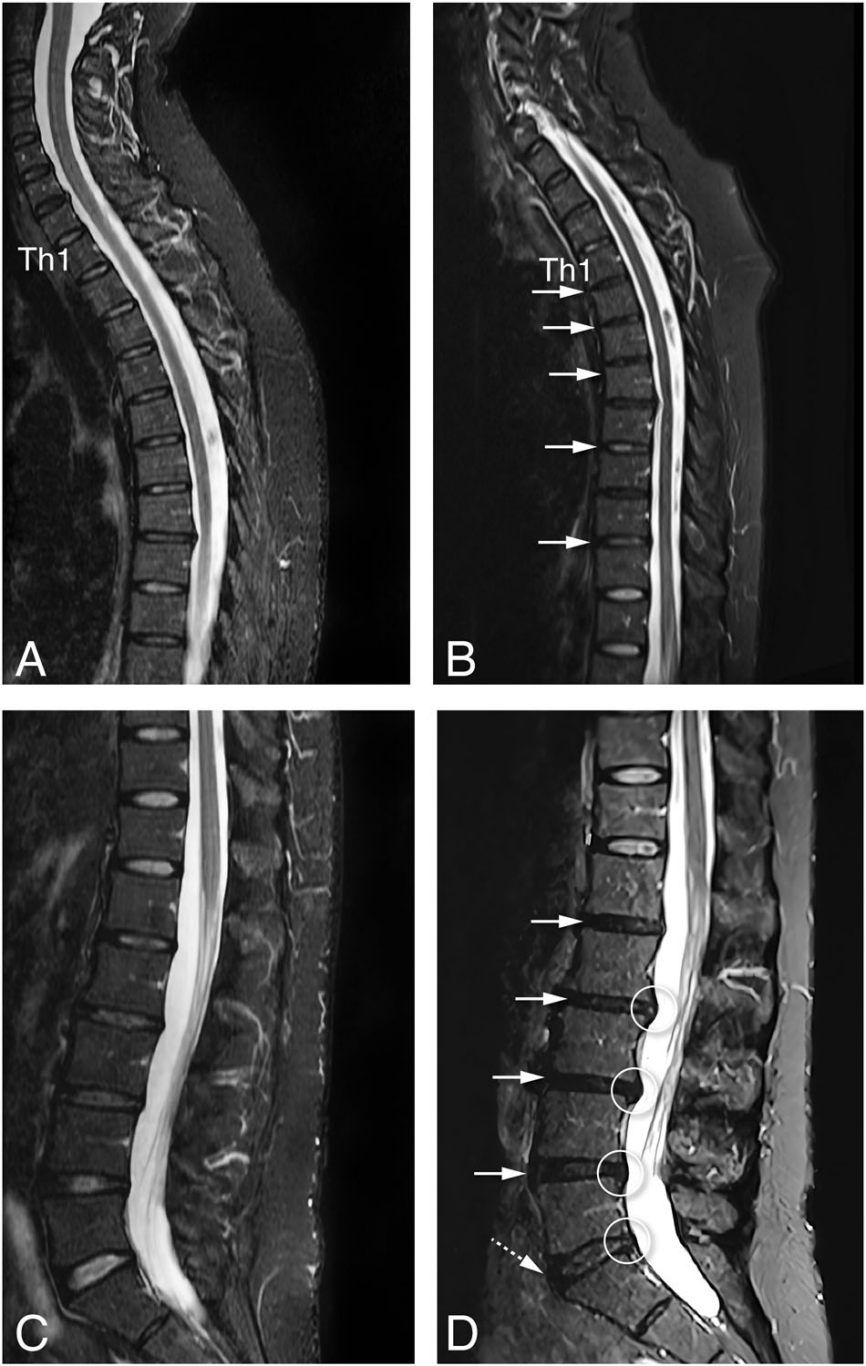
Example of a patient with a progression of DLs in the cervical, thoracic and lumbar spines on MRI over 10Y. Sagittal STIR images at baseline and 10-year follow-up of the cervicothoracic (**A**, **B**) and lumbar (**C**, **D**) spine. Decrease in height of the intervertebral disc spaces at multiple levels with a decrease of the signal of the discs Th1–Th4, Th5–Th6, and Th7–Th8 from grade II (normal discs) to grade IV (degenerated discs) (**A**, **B**). Progressive degenerative discs at all lumbar levels (white arrow) with the development of protrusion (circles) (**C**, **D**). L5-S1 (dashed arrow) progresses from grade II (normal disc) to grade III (degenerated disc)

At baseline, 267 (94%) patients had at least one DL on MRI, increasing to 275 (97%) at 10Y. The most frequent DLs on MRI were disc degeneration (Pfirrmann class > 2; baseline: 86%, 10Y: 95%, net progression: +9%), followed by HIZ (baseline: 50%, 10Y: 59%, net progression: +8%), Schmorl’s node without oedema (baseline: 47%, 10Y: 50%, net progression: +3%) and disc bulging/herniation (baseline: 46%, 10Y: 54%, net progression: +8%) (Table 2). No patient (0/53) at baseline and only 2% (2/123) at 10Y had osteophytes on radiographs without disc degeneration on MRI. An average total number of 7.4 (5.4) DLs per patient was observed on MRI at baseline; this number increased to 11.1 (7.1) at 10Y (*p* < 10^−4^) (Fig. 2). Similarly to radiographs, the total number of DLs increased with age.

**Table 2:** DLs observed on MRI at the patient level (at least one lesion in at least one vertebral level per patient) among patients with images available at baseline and at 10Y

	Baseline	5Y	10Y	Present at at least one time point	10-Year net progression
	*n* = 283	*n* = 113	*n* = 283	*n* = 283	*n* = 283
Pfirrmann class > 2	244 (86%)	102 (90%)	270 (95%)	272 (96%)	+9%
HIZ	142 (50%)	55 (49%)	166 (59%)	194 (68%)	+8%
Disc bulging/herniation	131 (46%)	62 (55%)	153 (54%)	181 (64%)	+8%
Schmorl’s node without oedema	133 (47%)	55 (49%)	141 (50%)	148 (52%)	+3%
Modic type I	16 (6%)	15 (13%)	39 (14%)	45 (16%)	+8%
Modic type II	12 (4%)	5 (4%)	25 (9%)	31 (11%)	+5%
Schmorl’s node with oedema	4 (1%)	2 (2%)	6 (2%)	10 (4%)	+1%
Lateral stenosis	2 (1%)	1 (1%)	8 (3%)	8 (3%)	+2%
CS	3 (1%)	3 (3%)	7 (2%)	7 (2%)	+1%
Spondylolisthesis	3 (1%)	5 (4%)	7 (2%)	7 (2%)	+1%
Modic type III	0 (0%)	0 (0%)	1 (0%)	1 (0%)	+0%
Scheuermann’s disease	1 (0%)	1 (1%)	1 (0%)	1 (0%)	0%
FJOA	0 (0%)	0 (0%)	0 (0%)	0 (0%)	0%
Number of DLs (mean (SD))	7.4 (5.4)	8.5 (6.2)	11.1 (7.1)	–	–
Patients aged < 30 years at baseline (*n* = 91)	5.1 (3.9)	5.7 (4.0)	7.5 (5.0)	–	–
Patients aged 30–40 years at baseline (*n* = 116)	7.3 (4.7)	7.9 (4.9)	11.4 (6.4)	–	–
Patients aged ≥ 40 years at baseline (*n* = 76)	10.4 (6.5)	11.7 (6.9)	15.0 (8.1)	–	–
Number of DLs in the cervical spine (mean (SD))	2.5 (2.4)	3.2 (2.6)	3.9 (2.7)	–	–
Number of DLs in the thoracic spine (mean (SD))	2.6 (3.1)	2.6 (3.1)	3.7 (3.9)	–	–
Number of DLs in the lumbar spine (mean (SD))	2.3 (2.2)	2.8 (2.6)	3.5 (3.0)	–	–
No DL	16 (6%)	4 (4%)	8 (3%)	–	–

Disc degeneration was scored on a five-point scale (Pfirrmann class), combining signal loss and loss of height of the intervertebral discs on STIR images. The HIZ, indicating an annular tear or fissure, is seen as an area of high signal intensity located in the posterior annulus fibrosis on STIR images. CS is defined as a reduction of the anterior–posterior diameter of the spinal canal with compression on the spinal cord (cervical/thoracic) or the cauda equina. Neural foraminal stenosis (lateral stenosis) was defined as contact between the nerve root and disc material with obliteration of perineural intraforaminal fat or compression of the nerve root. We considered discs extending beyond the limits of the anatomical disc space as disc bulging or herniation. FJOA was defined as sclerotic joint surfaces and osteophyte formation. Spondylolisthesis was defined as the slippage of one vertebral body with respect to the adjacent vertebral body. The Modic classification was used to assess DLs of the adjacent vertebral endplates. This is a three-point scale, with type I defined as bone marrow oedema (T1: low signal, STIR: high signal), type II defined as fatty changes (T1: high signal, STIR: low signal) and type III defined as sclerotic changes (T1: low signal, STIR: low signal). Schmorl’s nodes were defined as an indentation of the (cranial or caudal) endplate with herniation of intervertebral disc material into the vertebra, with or without oedema. Scheuermann’s disease was defined as abnormal and excessive curvature of the spin with anterior wedging of greater than or equal to 5 degrees in 3 or more adjacent vertebral bodies

*SD* standard deviation, *CS* canal stenosis, *FJOA* facet joint osteoarthritis, *DL* degenerative lesion

The total number of DLs was slightly higher in the lumbar than in the cervical spine on radiographs; they were evenly distributed over the whole spine on MRI. At baseline, 47 (16%) patients had both cervical and lumbar involvement on radiographs; this increased to 107 (37%) at 10Y. A significant association was found between these two locations (baseline: OR = 2.41, 95% CI = 1.44–4.03; 10Y: OR = 3.62, 2.22–5.91). Additionally, 34% (*n* = 96) initially had cervical and lumbar disc degeneration on MRI, rising to 57% (*n* = 160) after 10Y. A significant association was also observed for this modality (baseline: 1.91, 1.14–3.18; 10Y: 2.19, 1.18–4.07).

**Fig. 3:**
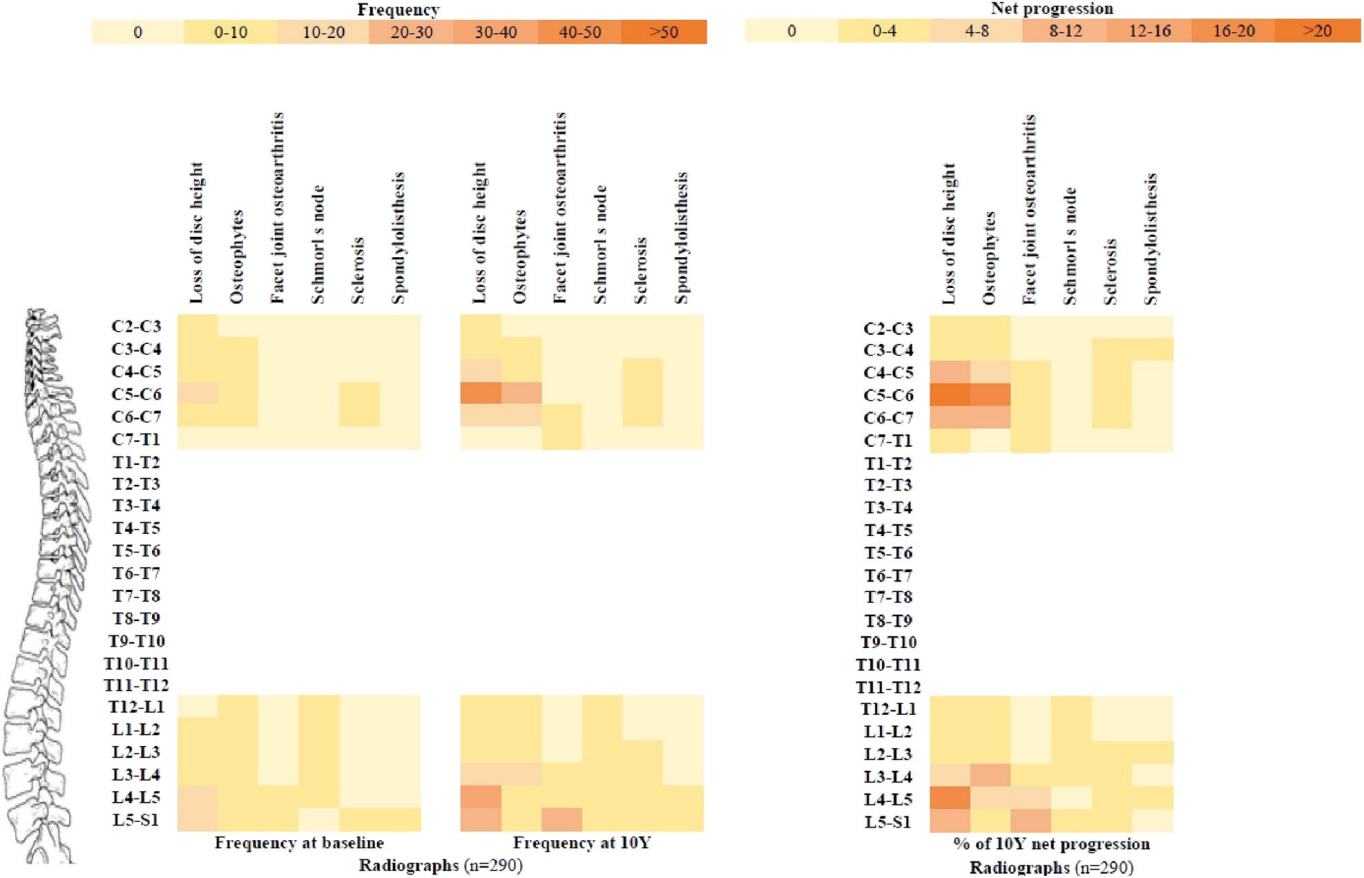
Heat maps of DL frequencies at baseline and 10Y and net progression over 10Y among patients with baseline and 10-year images at the vertebral unit level on radiographs. C, cervical; T, thoracic; L, lumbar; MRI, magnetic resonance imaging

**Fig. 4:**
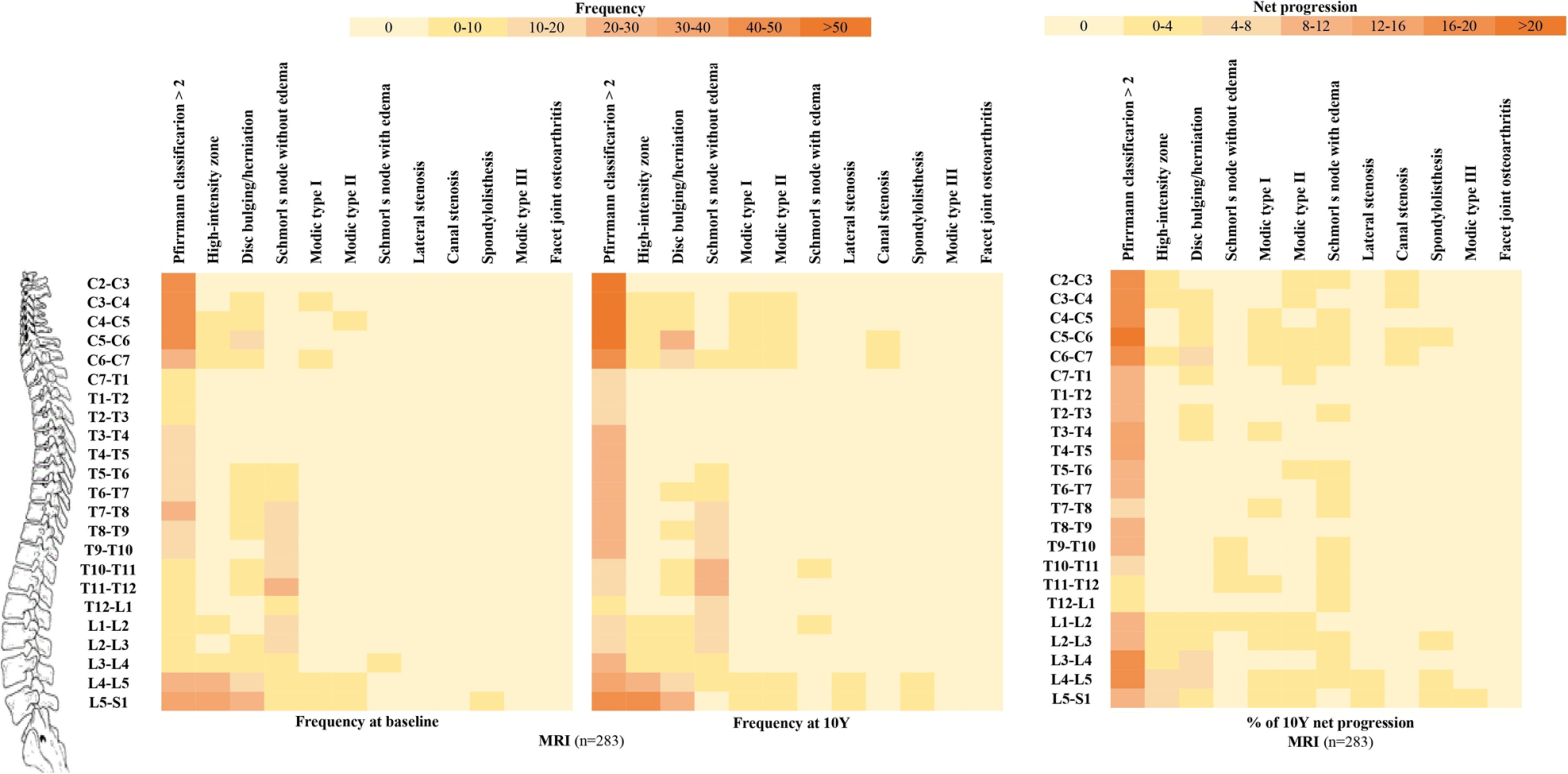
Heat maps of DL frequencies at baseline and 10Y and net progression over 10Y among patients with baseline and 10-year images at the vertebral unit level on MRI. C, cervical; T, thoracic; L, lumbar; MRI, magnetic resonance imaging

The distribution of each DL predominated in the lumbar spine in both imaging modalities, except for disc degeneration frequently observed in the cervical spine and Schmorl’s nodes frequently observed in the thoracic spine. The distribution of these spinal lesions at baseline and 10Y, and the 10Y net progression, are shown in the heat maps (Figs. 3 and 4). More detailed information on the distribution pattern of DLs in the whole spine at each time point is provided in Supplementary Tables S3–S8.

### Progression of degenerative spinal lesions over 10Y

After adjustment, a small annual progression was detected on radiographs mainly for osteophytes (annual change = 2.34%, 95% CI: 1.92–2.75), disc height loss (1.37%, 0.95–1.80), and FJOA (1.30%, 0.90–1.69). The same trend in annual progression was detected on MRI mainly for disc bulging/herniation (1.19%, 0.74–1.64), Modic type I (oedematous changes: 1.01%, 0.69–1.33), and II (fatty changes: 0.94%, 0.66–1.22) lesions, HIZ (0.90%, 0.45–1.35), and disc degeneration (0.89%, 0.63–1.15) (Table 3).

**Table 3:** Annual progression of DLs on radiographs and MRI

	% Annual change (95% CI)*
Progression on radiographs	*n* = 329
Loss of disc height	**1.37 (0.95; 1.80)**
Osteophytes	**2.34 (1.92; 2.75)**
FJOA	**1.30 (0.90; 1.69)**
Schmorl’s node	0.11 (−0.22; 0.43)
Sclerosis	**0.75 (0.51; 0.99)**
Spondylolisthesis	0.12 (−0.04; 0.28)
Progression on MRI	*n* = 327
Pfirrmann class > 2	**0.89 (0.63; 1.15)**
HIZ	**0.90 (0.45; 1.35)**
Disc bulging/herniation	**1.19 (0.74; 1.64)**
Schmorl’s node without oedema	0.11 (−0.01; 0.42)
Modic type I	**1.01 (0.69; 1.33)**
Modic type II	**0.94 (0.66; 1.22)**
Schmorl’s node with oedema	0.23 (−0.26; 0.64)
Lateral stenosis	**0.28 (0.12; 0.44)**
CS	**0.15 (0.01; 0.30)**
Spondylolisthesis	0.05 (−0.06; 0.16)
Modic type III	0.04 (−0.02; 0.10)
Scheuermann’s disease	0.01 (−0.07; 0.09)
FJOA	0.22 (−0.45; 0.88)

*MRI* magnetic resonance imaging, *95% CI* 95% confidence interval, *FJOA* facet joint osteoarthritis, *HIZ* high-intensity zone, *CS* canal stenosis

* Percentage change per year estimated used generalised estimating equation (GEE) models, considering individual reader data and exchangeable working correlation structure, adjusted for sex, BMI, HLA-B27 status, tobacco use, job type and bDMARDs exposure. Bold characters indicate significant values

### Factors associated with the progression of DLs

We observed a statistically significant annual increase in the number of DLs on radiographs (β = 0.18, 95% CI: 0.15–0.21) and MRI (β = 0.42,0.35–0.48) (Table 4). This increase was modest, with 1.8 lesions more on radiographs after 10Y. Factors associated with the increasing number of DLs over time in both imaging modalities were higher BMI (radiographs: β = 0.15, 0.12–0.19; MRI: β = 0.35, 0.27-0.44) and bDMARDs exposure (radiographs: β = 0.39, 0.13–0.66; MRI: β = 0.95, 0.34–1.55). The other associated factors are shown in Table 4.

**Table 4:** Factors associated with annual progression in the number of DLs on radiographs and MRI

	Radiographs *n* = 329	MRI *n* = 327
	β coefficients^*^ (95% CI)	
Number of DLs over time (/year)	**0.18 (0.15; 0.21)**	**0.42 (0.35; 0.48)**
Males (ref: women)	0.01 (−0.26; 0.28)	**1.36 (0.74; 1.99)**
BMI (kg/m^2^)	**0.15 (0.12; 0.19)**	**0.35 (0.27; 0.44)**
HLA-B27+	−**0.52 (−0.81; −0.22)**	−0.23 (−0.89; 0.43)
Smoking (ever vs never)	−0.03 (−0.31; 0.26)	−0.01 (−0.64; 0.62)
Job type (ref: blue collar)		
White collar	**0.62 (0.21; 1.02)**	0.21 (−0.74; 1.17)
Not employed	−0.40 (−0.93; 0.14)	−**2.40 (−3.59; −1.20)**
bDMARDs exposure (ever vs never)	**0.39 (0.13; 0.66)**	**0.95 (0.34; 1.55)**

Bold characters indicate significant values

*MRI* magnetic resonance imaging, *95% CI* 95% confidence interval, *BMI* body mass index, *HLA* human leucocyte antigen, *bDMARDs* biological disease-modifying antirheumatic drugs

* β Coefficients associated with annual progression estimated used generalised estimating equation (GEE) models, considering individual reader data and exchangeable working correlation structure

## Discussion

This study provides insight into the course of DLs throughout the spine on radiographs and MRI in an inception cohort of axSpA over a 10Y follow-up period. Their prevalence is high, even in these young patients, however, their change over 10Y is limited. The most common DL in the study was disc height loss on radiographs and disc degeneration on MRI. At baseline, the cervical and lumbar spines were predominantly involved. With ageing this increases, and the thoracic spine was also affected on MRI. The most significant DL progression concerned osteophytes on radiographs and disc bulging/herniation on MRI. Nevertheless, it remained modest, around 1% per year for most lesions, except for osteophytes, which showed the highest progression at 2% per year. Factors associated with an increase in the number of DLs over time in both modalities were increased BMI and bDMARDs exposure, the latter probably indicating a more severe form of axSpA.

This study is important because, to our knowledge, it is the first to assess spinal DL progression in a large population of young patients with axSpA on both radiographs and MRI. DLs are common in the spine, observed not only in subjects without axSpA [22–28], but also in patients with axSpA [2, 3, 29]. Although there are significant variations in the prevalence of DLs (differences in population age structure, and imaging acquisition methods, with most studies focusing only on the lumbar segment), those reported in this study appear to be in line with the prevalence published in the general population [22]. For example, in previous studies among patients under 50, lumbar disc degeneration ranged from 34% to 84%, and lumbar disc protrusion from 19% to 47% [26, 30], compared to 86% and 46% respectively in our study (baseline, whole spine). In a study involving 84 individuals (mean age: 38.5 years; 59% with chronic low back pain (LBP)) DLs were found in 52% on radiographs, similar to 53% in our study [28]. As observed here, the most frequent lesions in previous axSpA studies were disc height loss (21–34%) and osteophytes (13–26%) on radiographs; these were disc degeneration (42–63%), HIZ (23–65%) and Schmorl’s node without oedema (38–63%) on MRI [2, 3, 29]. These lesions predominated in the lumbar spine [29].

In a study of asymptomatic subjects, cervical disc degeneration progressed in 85% of individuals over 10Y on MRI [12]. Consistent with our study, the highest progression was reported for disc bulging/herniations. Thoracic disc degeneration on MRI progressed in 63% of subjects (mainly disc degeneration and protrusion) [31]. Similar observations have also been reported on radiographs [32]. In agreement with our results, disc degeneration progression also seems to affect mainly the lumbar spine [33, 34]. Recently, a long-term prospective follow-up of young patients with LBP demonstrated that the decrease in disc signal intensity on MRI at the age of 20 years was associated with any type of DLs in the same discs/disc levels at follow-up, suggesting a predictive value of this lesion for other types of DLs [33].

It should be borne in mind that despite the multitude of spinal DLs, their clinical significance may be limited [32]. Their presence is common even in patients without symptoms; conversely, some patients may suffer from back pain without any DL being observed [30, 31]. However, interestingly, patients with axSpA and coexisting disc degeneration have been reported to have higher functional and spinal mobility impairment [29].

We observed that DL progression was faster in patients with a higher BMI. Obesity is known to be associated with a higher prevalence of DLs in the general population [25, 26]. Although the exact mechanism is not well understood, this may be linked to a combination of mechanical (leading to higher compression and shear stresses), and metabolic (affecting the vascularisation of intervertebral discs) stresses [35]. This finding should encourage clinicians to emphasise the importance of weight loss in cases of overweight, including in a young axSpA population.

Limitations of this study include a substantial loss to follow-up rate (inception cohort), resulting in analysis of only part of the patients initially included in the cohort. Thoracic spine radiographs were unavailable, as per the DESIR protocol. However, DL assessment on radiographs is known to be limited, and this segment was explored using MRI. Additionally, since only sagittal MRI was performed, reliable FJOA scoring was not possible, and the assessment of disc herniation was limited. Of note, the progression of individual DLs, such as an increase in their size or extent over time, has not been assessed in this study, making it difficult to draw conclusions about the dynamic changes in specific lesions. In addition, although we used standardised imaging protocols, it is possible that the scanners underwent improvements between the beginning and end of the study. Finally, no data was available on the possible symptoms associated with the DLs observed.

This study has several strengths. The DESIR cohort is a multicentre study including a large number of well-phenotyped patients with high-quality clinical data spanning a decade-long follow-up period. DL evaluation by three central trained readers, able to discriminate DLs from axSpA lesions, provides robust data for comparison with those of the general population. The typical inflammatory lesions of axSpA mainly include anterior and posterior spondylitis (inflammatory corner lesions). However, other less specific inflammatory lesions, such as inflammatory spondylodiscitis, can be confused with certain DLs, such as Modic type 1 lesions (oedematous changes) [36]. Nevertheless, a previous study on the DESIR cohort showed that the number of vertebral units with overlap between the two types of lesions remained low, highlighting that differentiation is still possible in most cases [3]. Moreover, as this study was descriptive, it did not aim to assess the relationship between specific axSpA lesions and DLs and further analysis of these outcomes is currently underway. Finally, the use of statistical analyses tailored to the data structure (multilevel GEE models), sheds light on the DLs progression, which to our knowledge has not yet been performed.

In conclusion, the occurrence of spinal DLs is high in an inception cohort of axSpA with the DLs total number increasing over 10Y in the cervical and lumbar spine, and extending to the thoracic spine, on both radiographs and MRI. However, these lesions appear to progress slowly. In patients with a higher BMI or in those exposed to bDMARDs, which likely indicates a more severe form of axSpA, progression appears to be more rapid. Therefore, in axSpA, DLs are important to be borne in mind when interpreting imaging, particularly after several years of follow-up.

## Supplementary information

ELECTRONIC SUPPLEMENTARY MATERIAL

## Abbreviations

10Y: 10 Years
95% CI: 95% Confidence interval
ASAS: Assessment of spondyloArthritis International Society
axSpA: Axial spondyloarthritis
bDMARD: Biological disease-modifying antirheumatic drug
BMI: Body mass index
CS: Canal stenosis
DESIR: DEvenir des spondylarthropathies indifférenciées récentes
DL: Degenerative lesion
FJOA: Facet joint osteoarthritis
GEE: Generalised estimating equation
HIZ: High-intensity zone
HLA: Human leucocyte antigen
ICC: Intraclass correlation coefficient
LBP: Low back pain
MRI: Magnetic resonance imaging
OR: Odds ratio
Pfirrmann class: Pfirrmann classification
SD: Standard deviation
STIR: Short-tau inversion recovery
