# Supplementary material for: Ten-year follow-up of degenerative spinal lesions on radiographs and MRI in axial spondyloarthritis: results of the DESIR (DEvenir des spondylarthropathies indifférenciées récentes) cohort
Source: Eur Radiol. 2025 Mar 6;35(9):5381–91. doi: 10.1007/s00330-025-11432-4 (PMC12350588; doi:10.1007/s00330-025-11432-4)
Supplement: Supplementary file 1 — ELECTRONIC SUPPLEMENTARY MATERIAL [file 330_2025_11432_MOESM1_ESM.pdf]

Ten-year follow up of degenerative spinal lesions on radiographs and MRI in axial spondyloarthritis: results of the DESIR (DEvenir des Spondylarthropathies Indifférenciées Récentes) cohort

ELECTRONIC SUPPLEMENTARY MATERIAL

Summary

Supplementary Table S1. Baseline characteristics of the patients included	2
Supplementary Table S2. Inter-reader reliability on the total number of degenerative lesions at the patient level on radiographs and MRI	3
Supplementary Table S3. Degenerative lesions observed on radiographs at the vertebral unit level at baseline among patients with images available at baseline and at 10 years	4
Supplementary Table S4. Degenerative lesions observed on MRI at the vertebral unit level at baseline among patients with images available at baseline and at 10 years	5
Supplementary Table S5. Degenerative lesions observed on radiographs at the vertebral unit level at 5 years among patients with images available at baseline and at 10 years	7
Supplementary Table S6. Degenerative lesions observed on MRI at the vertebral unit level at 5 years among patients with images available at baseline and at 10 years	8
Supplementary Table S7. Degenerative lesions observed on radiographs at the vertebral unit level at 10 years among patients with images available at baseline and at 10 years	10
Supplementary Table S8. Degenerative lesions observed on MRI at the vertebral unit level at 10 years among patients with images available at baseline and at 10 years	11

Supplementary Table S1. Baseline characteristics of the patients included

**Patients**	**Overall** n=330
**Demographic characteristics**	
Age	34.5 (8.6)
Males	154 (47%)
Ethnicity (self-report)	
Asian	2 (1%)
Black African	7 (2%)
Caucasian	299 (91%)
Maghrebian	14 (4%)
Other°	8 (2%)
Education level	*(/329)*
Primary school	3 (0%)
Secondary school	114 (35%)
University for ≤ 3 years	113 (35%)
University for > 3 years	99 (30%)
Profession	*(/327)*
Blue collar*	46 (14%)
White collar**	246 (75%)
Not employed	35 (11%)
BMI (kg/m^2)	24.0 (4.1) *(/327)*
Smoking	117 (36%) *(/327)*
**Clinical characteristics**	
ASAS criteria (central reading)	214 (66%) *(/327)*
HLA-B27+	213 (64%)
History of dactylitis	46 (14%)
History of peripheral arthritis	94 (29%) *(/328)*
History of enthesitis	194 (59%)
Tender joints (/53 sites)	3.5 (6.6)
Swollen joints (/28 sites)	0.2 (1.0)
Enthesitis index (/13 sites: concise Mander Enthesitis score)	4.0 (5.4)
History of psoriasis	61 (19%)
History of IBD	18 (5%)
History of uveitis	31 (10%)
CRP at baseline (mg/L)	8.6 (14.1) *(/317)*
ASDAS	2.6 (1.0) *(/313)*
BASDAI (/100)	43.1 (20.4) *(/329)*
BASFI (/100)	28.7 (22.5) *(/326)*
Radiographs sacroiliitis (mNY criteria, central reading)	50 (16%) *(/320)*
MRI active sacroiliitis (ASAS criteria, central reading)	95 (30%) *(/319)*
**Treatment exposure (last 6 months)**	
NSAIDs	306 (93%)
Corticosteroids	45 (14%)
csDMARDs	48 (15%)

Data reported as n (%) or mean (Standard Deviation). Italics indicate the number of patients with available data.

°The sub-group is too small for meaningful analysis.

*farmer, craftsman or workmen; **business executive and intellectual profession, intermediate occupation, employee.

MRI: magnetic resonance imaging; BMI: body mass index; ASAS: Assessment of SpondyloArthritis international Society; SpA: *SpondyloArthritis; HLA: human leukocyte antigen; IBD: inflammatory bowel disease; CRP: C-reactive protein; ASDAS: Axial Spondyloarthritis Disease Activity Score; BASDAI: Bath Ankylosing Spondylitis Disease Activity Index; BASFI: Bath Ankylosing Spondylitis Functional Index; mNY: modified New York; NSAID: non-steroidal anti-inflammatory drug; csDMARD: conventional synthetic disease-modifying antirheumatic drug.*

Supplementary Table S2. Inter-reader reliability on the total number of degenerative lesions at the patient level on radiographs and MRI

	**Radiographs** n = 290			**MRI** n = 283		
	**Baseline**	**10Y**	**10Y change**	**Baseline**	**10Y**	**10Y change**
	**ICC (95%CI)**					
**Total number of degenerative lesions**	**0.60 (0.54-0.66)**	**0.74 (0.70-0.78)**	**0.51 (0.44-0.58)**	**0.69 (0.65-0.74)**	**0.76 (0.70-0.79)**	**0.49 (0.42-0.56)**

Bold characters indicate significant values.

MRI: Magnetic Resonance Imaging; ICC: Intraclass Correlation Coefficients; 95%CI: 95% confidence interval; Y: years.

**ICC**			
<0.40	0.40-	0.60-	≥0.75

Supplementary Table S3. Degenerative lesions observed on radiographs at the vertebral unit level at baseline in patients with images available at baseline and at 10 years

	Radiographs n = 290					
	Loss of disc height	Osteophytes	FJOA	Schmorl's node	Sclerosis	Spondylolisthesis
**C2-C3**	4 (1%)	0 (0%)	0 (0%)	0 (0%)	0 (0%)	0 (0%)
**C3-C4**	5 (2%)	5 (2%)	1 (0%)	0 (0%)	0 (0%)	0 (0%)
**C4-C5**	28 (10%)	11 (4%)	0 (0%)	0 (0%)	0 (0%)	0 (0%)
**C5-C6**	56 (19%)	28 (10%)	0 (0%)	0 (0%)	2 (1%)	0 (0%)
**C6-C7**	25 (9%)	15 (5%)	1 (0%)	0 (0%)	4 (1%)	0 (0%)
**C7-T1**	0 (0%)	0 (0%)	1 (0%)	0 (0%)	0 (0%)	0 (0%)
**T12-L1**	0 (0%)	4 (1%)	0 (0%)	7 (2%)	0 (0%)	0 (0%)
**L1-L2**	4 (1%)	6 (2%)	0 (0%)	12 (4%)	1 (0%)	0 (0%)
**L2-L3**	10 (3%)	12 (4%)	0 (0%)	11 (4%)	1 (0%)	0 (0%)
**L3-L4**	19 (7%)	19 (7%)	0 (0%)	3 (1%)	0 (0%)	0 (0%)
**L4-L5**	51 (18%)	10 (3%)	7 (2%)	2 (1%)	1 (0%)	1 (0%)
**L5-S1**	50 (17%)	6 (2%)	27 (9%)	1 (0%)	2 (1%)	4 (1%)

FJOA: facet joint osteoarthritis.

Frequency						
0%	0-10%	10-20%	20-30%	30-40%	40-50%	>50%

Supplementary Table S4. Degenerative lesions observed on MRI at the vertebral unit level at baseline in patients with images available at baseline and at 10 years

	MRI n = 283															
	Pfirrmann class.: grade 1	Pfirrmann class.: grade 2	Pfirrmann class.: grade 3	Pfirrmann class.: grade 4	Pfirrmann class.: grade 5	Pfirrmann class. > 2	HIZ	Disc bulging / herniation	Schmorl's node without edema	Modic type I	Schmorl's node with edema	Lateral/ neuroforaminal CS	Central CS	Spondylo-listhesis	Modic type III	FJOA
**C2-C3**	33 (12%)	115 (41%)	113 (40%)	6 (2%)	0 (0%)	119 (42%)	0 (0%)	0 (0%)	0 (0%)	1 (0%)	0 (0%)	0 (0%)	0 (0%)	0 (0%)	0 (0%)	0 (0%)
**C3-C4**	28 (10%)	112 (40%)	119 (42%)	13 (5%)	0 (0%)	132 (47%)	1 (0%)	12 (4%)	0 (0%)	2 (1%)	0 (0%)	0 (0%)	0 (0%)	0 (0%)	0 (0%)	0 (0%)
**C4-C5**	34 (12%)	114 (40%)	105 (37%)	13 (5%)	1 (0%)	119 (42%)	4 (1%)	14 (5%)	0 (0%)	0 (0%)	0 (0%)	0 (0%)	1 (0%)	0 (0%)	0 (0%)	0 (0%)
**C5-C6**	34 (12%)	100 (35%)	110 (39%)	18 (6%)	3 (1%)	131 (46%)	11 (4%)	40 (14%)	0 (0%)	1 (0%)	0 (0%)	0 (0%)	1 (0%)	0 (0%)	0 (0%)	0 (0%)
**C6-C7**	79 (28%)	112 (40%)	64 (23%)	9 (3%)	2 (1%)	75 (27%)	4 (1%)	20 (7%)	1 (0%)	3 (1%)	0 (0%)	0 (0%)	1 (0%)	0 (0%)	0 (0%)	0 (0%)
**C7-T1**	133 (47%)	122 (43%)	14 (5%)	1 (0%)	0 (0%)	15 (5%)	0 (0%)	0 (0%)	0 (0%)	0 (0%)	0 (0%)	0 (0%)	0 (0%)	0 (0%)	0 (0%)	0 (0%)
**T1-T2**	121 (43%)	133 (47%)	20 (7%)	1 (0%)	0 (0%)	21 (7%)	0 (0%)	0 (0%)	0 (0%)	0 (0%)	0 (0%)	0 (0%)	0 (0%)	0 (0%)	0 (0%)	0 (0%)
**T2-T3**	105 (37%)	151 (53%)	20 (7%)	2 (1%)	1 (0%)	23 (8%)	0 (0%)	0 (0%)	0 (0%)	0 (0%)	0 (0%)	0 (0%)	0 (0%)	0 (0%)	0 (0%)	0 (0%)
**T3-T4**	97 (34%)	145 (51%)	27 (10%)	4 (1%)	0 (0%)	31 (11%)	0 (0%)	0 (0%)	0 (0%)	0 (0%)	0 (0%)	0 (0%)	0 (0%)	0 (0%)	0 (0%)	0 (0%)
**T4-T5**	100 (35%)	135 (48%)	26 (9%)	8 (3%)	1 (0%)	35 (12%)	0 (0%)	1 (0%)	1 (0%)	0 (0%)	0 (0%)	0 (0%)	0 (0%)	0 (0%)	0 (0%)	0 (0%)
**T5-T6**	93 (33%)	128 (45%)	27 (10%)	15 (5%)	0 (0%)	42 (15%)	0 (0%)	2 (1%)	6 (2%)	0 (0%)	0 (0%)	0 (0%)	0 (0%)	0 (0%)	0 (0%)	0 (0%)
**T6-T7**	90 (32%)	117 (41%)	37 (13%)	18 (6%)	0 (0%)	55 (19%)	1 (0%)	2 (1%)	26 (9%)	0 (0%)	1 (0%)	0 (0%)	0 (0%)	0 (0%)	0 (0%)	0 (0%)
**T7-T8**	89 (31%)	116 (41%)	48 (17%)	10 (4%)	1 (0%)	59 (21%)	0 (0%)	2 (1%)	41 (14%)	0 (0%)	0 (0%)	0 (0%)	0 (0%)	0 (0%)	0 (0%)	0 (0%)
**T8-T9**	83 (29%)	124 (44%)	50 (18%)	7 (2%)	1 (0%)	58 (20%)	0 (0%)	2 (1%)	44 (15%)	0 (0%)	0 (0%)	0 (0%)	0 (0%)	0 (0%)	0 (0%)	0 (0%)
**T9-T10**	75 (27%)	143 (54%)	28 (10%)	7 (2%)	2 (1%)	37 (13%)	1 (0%)	0 (0%)	50 (18%)	0 (0%)	0 (0%)	0 (0%)	0 (0%)	0 (0%)	0 (0%)	0 (0%)
**T10-T11**	66	185	21	2	0	23	0	2	53	0	0	0	0	0	0	0

	(23%)	(65%)	(7%)	(1%)	(0%)	(8%)	(0%)	(1%)	(19%)	(0%)	(0%)	(0%)	(0%)	(0%)	(0%)	(0%)
**T11-T12**	35 (12%)	211 (75%)	19 (7%)	6 (2%)	0 (0%)	25 (9%)	1 (0%)	3 (1%)	73 (26%)	0 (0%)	0 (0%)	0 (0%)	0 (0%)	0 (0%)	0 (0%)	0 (0%)
**T12-L1**	23 (8%)	243 (86%)	12 (4%)	1 (0%)	0 (0%)	13 (4%)	0 (0%)	0 (0%)	29 (10%)	0 (0%)	0 (0%)	0 (0%)	0 (0%)	0 (0%)	0 (0%)	0 (0%)
**L1-L2**	7 (2%)	261 (92%)	10 (4%)	2 (1%)	0 (0%)	12 (4%)	2 (1%)	1 (0%)	43 (15%)	0 (0%)	1 (0%)	0 (0%)	0 (0%)	0 (0%)	0 (0%)	0 (0%)
**L2-L3**	11 (4%)	252 (89%)	14 (5%)	2 (1%)	1 (0%)	17 (6%)	1 (0%)	2 (1%)	31 (11%)	0 (0%)	1 (0%)	0 (0%)	0 (0%)	0 (0%)	0 (0%)	0 (0%)
**L3-L4**	17 (6%)	235 (83%)	21 (7%)	3 (1%)	0 (0%)	24 (8%)	7 (2%)	5 (2%)	17 (6%)	1 (0%)	2 (1%)	0 (0%)	0 (0%)	0 (0%)	0 (0%)	0 (0%)
**L4-L5**	19 (7%)	191 (67%)	49 (17%)	12 (4%)	1 (0%)	62 (22%)	59 (21%)	32 (11%)	12 (4%)	4 (1%)	0 (0%)	1 (0%)	1 (0%)	1 (0%)	0 (0%)	0 (0%)
**L5-S1**	17 (6%)	164 (58%)	56 (20%)	31 (11%)	4 (1%)	91 (32%)	107 (38%)	63 (22%)	2 (1%)	7 (2%)	0 (0%)	1 (0%)	1 (0%)	2 (1%)	0 (0%)	0 (0%)

MRI: Magnetic Resonance Imaging; Pfirrmann class.: Pfirrmann classification; HIZ: high-intensity zone; CS: canal stenosis; FJOA: facet joint osteoarthritis.

Frequency

0%	0-10%	10-20%	20-30%	30-40%	40-50%	>50%

Supplementary Table S5. Degenerative lesions observed on radiographs at the vertebral unit level at 5 years in patients with images available at baseline and at 10 years

	Radiographs n = 258					
	Loss of disc height	Osteophytes	FJOA	Schmorl's node	Sclerosis	Spondylolisthesis
**C2-C3**	4 (2%)	0 (0%)	0 (0%)	0 (0%)	0 (0%)	0 (0%)
**C3-C4**	4 (2%)	4 (2%)	0 (0%)	0 (0%)	0 (0%)	0 (0%)
**C4-C5**	40 (16%)	16 (6%)	1 (0%)	0 (0%)	1 (0%)	0 (0%)
**C5-C6**	74 (29%)	36 (14%)	0 (0%)	0 (0%)	3 (1%)	0 (0%)
**C6-C7**	35 (14%)	31 (12%)	1 (0%)	0 (0%)	5 (2%)	0 (0%)
**C7-T1**	0 (0%)	0 (0%)	2 (1%)	0 (0%)	0 (0%)	0 (0%)
**T12-L1**	2 (1%)	5 (2%)	0 (0%)	7 (2%)	0 (0%)	0 (0%)
**L1-L2**	9 (3%)	9 (3%)	0 (0%)	12 (4%)	1 (0%)	0 (0%)
**L2-L3**	14 (5%)	16 (6%)	0 (0%)	11 (4%)	3 (1%)	1 (0%)
**L3-L4**	24 (9%)	30 (12%)	1 (0%)	3 (1%)	2 (1%)	0 (0%)
**L4-L5**	64 (25%)	17 (7%)	18 (7%)	2 (1%)	2 (1%)	4 (2%)
**L5-S1**	58 (22%)	10 (4%)	39 (15%)	1 (0%)	6 (2%)	1 (0%)

FJOA: facet joint osteoarthritis.

Frequency

0%	0-10%	10-20%	20-30%	30-40%	40-50%	>50%

Supplementary Table S6. Degenerative lesions observed on MRI at the vertebral unit level at 5 years in patients with images available at baseline and at 10 years

	MRI n = 113																
	Pfirrmann class.: grade 1	Pfirrmann class.: grade 2	Pfirrmann class.: grade 3	Pfirrmann class.: grade 4	Pfirrmann class.: grade 5	Pfirrmann class. > 2	HIZ	Disc bulging / herniation	Schmorl's node without edema	Modic type I	Modic type II	Schmorl's node with edema	Lateral/ neurofora minal CS	Central CS	Spondylo -listhesis	Modic type III	FJOA
**C2-C3**	11 (10%)	39 (45%)	56 (50%)	3 (3%)	0 (0%)	59 (52%)	0 (0%)	0 (0%)	0 (0%)	0 (0%)	0 (0%)	0 (0%)	0 (0%)	0 (0%)	0 (0%)	0 (0%)	0 (0%)
**C3-C4**	11 (10%)	33 (29%)	59 (52%)	4 (4%)	0 (0%)	63 (56%)	2 (2%)	7 (6%)	0 (0%)	0 (0%)	1 (1%)	0 (0%)	0 (0%)	0 (0%)	0 (0%)	0 (0%)	0 (0%)
**C4-C5**	12 (11%)	40 (35%)	46 (41%)	8 (7%)	1 (1%)	55 (49%)	4 (4%)	13 (12%)	0 (0%)	0 (0%)	0 (0%)	0 (0%)	0 (0%)	2 (2%)	0 (0%)	0 (0%)	0 (0%)
**C5-C6**	12 (11%)	30 (27%)	50 (44%)	8 (7%)	2 (2%)	60 (53%)	3 (3%)	19 (17%)	0 (0%)	4 (4%)	2 (2%)	0 (0%)	0 (0%)	1 (1%)	0 (0%)	0 (0%)	0 (0%)
**C6-C7**	35 (31%)	39 (35%)	25 (22%)	6 (5%)	1 (1%)	32 (28%)	0 (0%)	14 (12%)	1 (1%)	1 (1%)	0 (0%)	0 (0%)	0 (0%)	2 (2%)	0 (0%)	0 (0%)	0 (0%)
**C7-T1**	57 (50%)	40 (35%)	11 (10%)	1 (1%)	0 (0%)	12 (11%)	0 (0%)	1 (1%)	0 (0%)	1 (1%)	0 (0%)	0 (0%)	0 (0%)	0 (0%)	0 (0%)	0 (0%)	0 (0%)
**T1-T2**	52 (46%)	42 (37%)	13 (12%)	1 (1%)	0 (0%)	14 (12%)	0 (0%)	0 (0%)	0 (0%)	0 (0%)	0 (0%)	0 (0%)	0 (0%)	0 (0%)	0 (0%)	0 (0%)	0 (0%)
**T2-T3**	44 (39%)	45 (40%)	15 (13%)	2 (2%)	1 (1%)	18 (16%)	0 (0%)	1 (1%)	0 (0%)	0 (0%)	0 (0%)	0 (0%)	0 (0%)	0 (0%)	0 (0%)	0 (0%)	0 (0%)
**T3-T4**	40 (35%)	50 (44%)	14 (12%)	3 (3%)	1 (1%)	18 (16%)	0 (0%)	1 (1%)	0 (0%)	0 (0%)	0 (0%)	0 (0%)	0 (0%)	0 (0%)	0 (0%)	0 (0%)	0 (0%)
**T4-T5**	41 (36%)	48 (42%)	9 (8%)	5 (4%)	1 (1%)	15 (13%)	0 (0%)	0 (0%)	0 (0%)	0 (0%)	0 (0%)	0 (0%)	0 (0%)	0 (0%)	0 (0%)	0 (0%)	0 (0%)
**T5-T6**	47 (41%)	41 (36%)	11 (10%)	6 (5%)	0 (0%)	17 (15%)	0 (0%)	1 (1%)	1 (1%)	0 (0%)	0 (0%)	0 (0%)	0 (0%)	0 (0%)	0 (0%)	0 (0%)	0 (0%)
**T6-T7**	46 (41%)	37 (33%)	18 (16%)	8 (7%)	0 (0%)	26 (23%)	0 (0%)	0 (0%)	11 (9%)	0 (0%)	0 (0%)	0 (0%)	0 (0%)	0 (0%)	0 (0%)	0 (0%)	0 (0%)
**T7-T8**	43 (38%)	41 (36%)	19 (17%)	4 (4%)	0 (0%)	23 (20%)	0 (0%)	1 (1%)	14 (12%)	0 (0%)	0 (0%)	0 (0%)	0 (0%)	0 (0%)	0 (0%)	0 (0%)	0 (0%)
**T8-T9**	37 (33%)	45 (40%)	18 (16%)	1 (1%)	0 (0%)	19 (17%)	0 (0%)	0 (0%)	13 (12%)	0 (0%)	0 (0%)	0 (0%)	0 (0%)	0 (0%)	0 (0%)	0 (0%)	0 (0%)
**T9-T10**	34 (30%)	59 (52%)	12 (11%)	1 (1%)	1 (1%)	14 (12%)	0 (0%)	0 (0%)	15 (13%)	0 (0%)	0 (0%)	1 (1%)	0 (0%)	0 (0%)	0 (0%)	0 (0%)	0 (0%)
**T10-T11**	30	71	6	0	0	6	0	0	19	0	0	0	0	0	0	0	0

	(27%)	(63%)	(5%)	(0%)	(0%)	(5%)	(0%)	(0%)	(17%)	(0%)	(0%)	(0%)	(0%)	(0%)	(0%)	(0%)	(0%)
**T11-T12**	17 (15%)	85 (75%)	7 (6%)	3 (3%)	0 (0%)	10 (9%)	0 (0%)	0 (0%)	30 (27%)	1 (1%)	0 (0%)	0 (0%)	0 (0%)	0 (0%)	0 (0%)	0 (0%)	0 (0%)
**T12-L1**	8 (7%)	94 (83%)	7 (6%)	1 (1%)	0 (0%)	8 (7%)	0 (0%)	1 (1%)	9 (8%)	0 (0%)	0 (0%)	0 (0%)	0 (0%)	0 (0%)	0 (0%)	0 (0%)	0 (0%)
**L1-L2**	4 (4%)	98 (87%)	8 (7%)	1 (1%)	0 (0%)	9 (8%)	1 (1%)	1 (1%)	12 (11%)	0 (0%)	1 (1%)	0 (0%)	0 (0%)	0 (0%)	0 (0%)	0 (0%)	0 (0%)
**L2-L3**	7 (6%)	95 (84%)	9 (8%)	0 (0%)	0 (0%)	9 (8%)	0 (0%)	2 (2%)	14 (12%)	0 (0%)	0 (0%)	0 (0%)	0 (0%)	0 (0%)	0 (0%)	0 (0%)	0 (0%)
**L3-L4**	10 (9%)	86 (76%)	13 (12%)	2 (2%)	1 (1%)	16 (14%)	4 (4%)	8 (7%)	11 (10%)	1 (1%)	0 (0%)	1 (1%)	0 (0%)	0 (0%)	1 (1%)	0 (0%)	0 (0%)
**L4-L5**	7 (6%)	70 (61%)	28 (25%)	3 (3%)	3 (3%)	34 (30%)	20 (18%)	15 (13%)	8 (7%)	5 (4%)	1 (1%)	0 (0%)	1 (1%)	0 (0%)	3 (3%)	0 (0%)	0 (0%)
**L5-S1**	7 (6%)	62 (55%)	24 (21%)	15 (13%)	4 (4%)	43 (38%)	40 (35%)	26 (23%)	1 (1%)	4 (4%)	2 (2%)	0 (0%)	0 (0%)	0 (0%)	1 (1%)	0 (0%)	0 (0%)

MRI: Magnetic Resonance Imaging; Pfirrmann class.: Pfirrmann classification; HIZ: high-intensity zone; CS: canal stenosis; FJOA: facet joint osteoarthritis.

Frequency

0%	0-10%	10-20%	20-30%	30-40%	40-50%	>50%

Supplementary Table S7. Degenerative lesions observed on radiographs at the vertebral unit level at 10 years in patients with images available at baseline and at 10 years

	Radiographs n = 290					
	Loss of disc height	Osteophytes	FJOA	Schmorl's node	Sclerosis	Spondylolisthesis
**C2-C3**	5 (2%)	1 (0%)	0 (0%)	0 (0%)	0 (0%)	0 (0%)
**C3-C4**	17 (6%)	10 (3%)	1 (0%)	0 (0%)	1 (0%)	1 (0%)
**C4-C5**	57 (20%)	27 (9%)	1 (0%)	0 (0%)	3 (1%)	0 (0%)
**C5-C6**	123 (42%)	70 (24%)	1 (0%)	0 (0%)	9 (3%)	0 (0%)
**C6-C7**	58 (20%)	45 (16%)	2 (1%)	0 (0%)	7 (2%)	0 (0%)
**C7-T1**	1 (0%)	0 (0%)	3 (1%)	0 (0%)	0 (0%)	0 (0%)
**T12-L1**	2 (1%)	8 (3%)	0 (0%)	9 (3%)	0 (0%)	0 (0%)
**L1-L2**	15 (5%)	15 (5%)	0 (0%)	18 (6%)	0 (0%)	0 (0%)
**L2-L3**	21 (7%)	24 (8%)	0 (0%)	13 (4%)	3 (1%)	1 (0%)
**L3-L4**	38 (13%)	44 (15%)	4 (1%)	5 (2%)	2 (1%)	0 (0%)
**L4-L5**	94 (32%)	27 (9%)	27 (9%)	2 (1%)	2 (1%)	7 (2%)
**L5-S1**	79 (27%)	17 (6%)	62 (21%)	2 (1%)	9 (3%)	2 (1%)

FJOA: facet joint osteoarthritis.

Frequency

0%	0-10%	10-20%	20-30%	30-40%	40-50%	>50%

Supplementary Table S8. Degenerative lesions observed on MRI at the vertebral unit level at 10 years in patients with images available at baseline and at 10 years

	MRI n = 283																
	Pfirrmann class.: grade 1	Pfirrmann class.: grade 2	Pfirrmann class.: grade 3	Pfirrmann class.: grade 4	Pfirrmann class.: grade 5	Pfirrmann class. > 2	HIZ	Disc bulging / herniation	Schmorl's node without edema	Modic type I	Modic type II	Schmorl's node with edema	Lateral/ neuroforaminal CS	Central CS	Spondylo-listhesis	Modic type III	FJOA
**C2-C3**	17 (6%)	77 (27%)	155 (55%)	13 (5%)	0 (0%)	168 (59%)	1 (0%)	0 (0%)	1 (0%)	1 (0%)	1 (0%)	0 (0%)	0 (0%)	0 (0%)	0 (0%)	0 (0%)	0 (0%)
**C3-C4**	11 (4%)	67 (24%)	164 (58%)	21 (7%)	1 (0%)	186 (66%)	2 (1%)	16 (6%)	0 (0%)	2 (1%)	2 (1%)	0 (0%)	0 (0%)	1 (0%)	0 (0%)	0 (0%)	0 (0%)
**C4-C5**	14 (5%)	69 (24%)	140 (50%)	27 (10%)	5 (2%)	172 (61%)	4 (1%)	28 (10%)	1 (0%)	5 (2%)	2 (1%)	0 (0%)	0 (0%)	1 (0%)	0 (0%)	0 (0%)	0 (0%)
**C5-C6**	16 (6%)	47 (17%)	140 (50%)	42 (15%)	8 (3%)	190 (67%)	10 (4%)	57 (20%)	1 (0%)	12 (4%)	5 (2%)	0 (0%)	0 (0%)	2 (1%)	1 (0%)	0 (0%)	0 (0%)
**C6-C7**	45 (16%)	85 (30%)	86 (30%)	26 (9%)	7 (2%)	117 (42%)	5 (2%)	36 (13%)	2 (1%)	10 (4%)	3 (1%)	0 (0%)	0 (0%)	3 (1%)	0 (0%)	0 (0%)	0 (0%)
**C7-T1**	71 (25%)	154 (54%)	37 (13%)	5 (2%)	0 (0%)	42 (15%)	0 (0%)	1 (0%)	0 (0%)	0 (0%)	1 (0%)	0 (0%)	0 (0%)	0 (0%)	0 (0%)	0 (0%)	0 (0%)
**T1-T2**	62 (22%)	156 (55%)	44 (16%)	6 (2%)	0 (0%)	50 (18%)	0 (0%)	0 (0%)	0 (0%)	0 (0%)	0 (0%)	0 (0%)	0 (0%)	0 (0%)	0 (0%)	0 (0%)	0 (0%)
**T2-T3**	62 (22%)	145 (51%)	51 (18%)	6 (2%)	1 (0%)	58 (20%)	0 (0%)	1 (0%)	1 (0%)	0 (0%)	0 (0%)	0 (0%)	0 (0%)	0 (0%)	0 (0%)	0 (0%)	0 (0%)
**T3-T4**	62 (22%)	131 (46%)	62 (22%)	9 (3%)	1 (0%)	72 (25%)	0 (0%)	1 (0%)	0 (0%)	1 (0%)	0 (0%)	0 (0%)	0 (0%)	0 (0%)	0 (0%)	0 (0%)	0 (0%)
**T4-T5**	67 (24%)	112 (40%)	59 (21%)	15 (5%)	3 (1%)	77 (27%)	0 (0%)	1 (0%)	1 (0%)	0 (0%)	0 (0%)	0 (0%)	0 (0%)	0 (0%)	0 (0%)	0 (0%)	0 (0%)
**T5-T6**	67 (24%)	107 (38%)	60 (21%)	17 (6%)	0 (0%)	77 (27%)	0 (0%)	1 (0%)	7 (2%)	0 (0%)	1 (0%)	0 (0%)	0 (0%)	0 (0%)	0 (0%)	0 (0%)	0 (0%)
**T6-T7**	65 (23%)	114 (40%)	54 (19%)	30 (11%)	2 (1%)	86 (30%)	1 (0%)	2 (1%)	27 (10%)	0 (0%)	0 (0%)	0 (0%)	0 (0%)	0 (0%)	0 (0%)	0 (0%)	0 (0%)
**T7-T8**	72 (25%)	106 (37%)	59 (21%)	22 (8%)	2 (1%)	83 (29%)	0 (0%)	1 (0%)	46 (16%)	1 (0%)	0 (0%)	0 (0%)	0 (0%)	0 (0%)	0 (0%)	0 (0%)	0 (0%)
**T8-T9**	67 (24%)	113 (40%)	65 (23%)	15 (5%)	3 (1%)	83 (29%)	0 (0%)	2 (1%)	44 (16%)	0 (0%)	0 (0%)	0 (0%)	0 (0%)	0 (0%)	0 (0%)	0 (0%)	0 (0%)
**T9-T10**	58 (21%)	142 (50%)	46 (16%)	14 (5%)	3 (1%)	63 (22%)	0 (0%)	0 (0%)	53 (19%)	0 (0%)	0 (0%)	1 (0%)	0 (0%)	0 (0%)	0 (0%)	0 (0%)	0 (0%)
**T10-T11**	45	181	32	7	0	39	0	2	61	0	0	2	0	0	0	0	0

	(16%)	(64%)	(11%)	(2%)	(0%)	(14%)	(0%)	(1%)	(22%)	(0%)	(0%)	(1%)	(0%)	(0%)	(0%)	(0%)	(0%)
**T11-T12**	23 (8%)	210 (74%)	24 (8%)	9 (3%)	0 (0%)	33 (12%)	1 (0%)	2 (1%)	76 (27%)	1 (0%)	1 (0%)	1 (0%)	0 (0%)	0 (0%)	0 (0%)	0 (0%)	0 (0%)
**T12-L1**	11 (4%)	246 (87%)	17 (6%)	1 (0%)	1 (0%)	19 (7%)	0 (0%)	1 (0%)	32 (11%)	0 (0%)	0 (0%)	0 (0%)	0 (0%)	0 (0%)	0 (0%)	0 (0%)	0 (0%)
**L1-L2**	2 (1%)	237 (84%)	30 (11%)	8 (3%)	0 (0%)	38 (13%)	4 (1%)	5 (2%)	42 (15%)	1 (0%)	1 (0%)	2 (1%)	0 (0%)	0 (0%)	0 (0%)	0 (0%)	0 (0%)
**L2-L3**	2 (1%)	228 (81%)	39 (14%)	8 (3%)	1 (0%)	48 (17%)	8 (3%)	11 (4%)	33 (12%)	1 (0%)	1 (0%)	1 (0%)	0 (0%)	0 (0%)	1 (0%)	0 (0%)	0 (0%)
**L3-L4**	3 (1%)	205 (72%)	56 (20%)	11 (4%)	1 (0%)	68 (24%)	14 (5%)	19 (7%)	20 (7%)	1 (0%)	0 (0%)	1 (0%)	0 (0%)	0 (0%)	0 (0%)	0 (0%)	0 (0%)
**L4-L5**	4 (1%)	151 (53%)	85 (30%)	22 (8%)	3 (1%)	110 (39%)	76 (27%)	49 (17%)	13 (5%)	7 (2%)	3 (1%)	0 (0%)	5 (2%)	0 (0%)	3 (1%)	0 (0%)	0 (0%)
**L5-S1**	7 (2%)	145 (51%)	73 (26%)	39 (14%)	11 (4%)	123 (43%)	123 (43%)	73 (26%)	1 (0%)	9 (3%)	10 (4%)	0 (0%)	4 (1%)	0 (0%)	3 (1%)	1 (0%)	0 (0%)

MRI: Magnetic Resonance Imaging; Pfirrmann class.: Pfirrmann classification; HIZ: high-intensity zone; CS: canal stenosis; FJOA: facet joint osteoarthritis.

Frequency

0%	0-10%	10-20%	20-30%	30-40%	40-50%	>50%
